# Impacts of Wet Market Modernization Levels and Hygiene Practices on the Microbiome and Microbial Safety of Wooden Cutting Boards in Hong Kong

**DOI:** 10.3390/microorganisms8121941

**Published:** 2020-12-07

**Authors:** Wing Yui Ngan, Subramanya Rao, Long Chung Chan, Patrick T. Sekoai, Yang Pu, Yuan Yao, Aster Hei Yiu Fung, Olivier Habimana

**Affiliations:** The School of Biological Sciences, The University of Hong Kong, Pokfulam, Hong Kong, China; ngan@evolbio.mpg.de (W.Y.N.); subbu36@gmail.com (S.R.); kodachi@connect.hku.hk (L.C.C.); patricksekoai@gmail.com (P.T.S.); puyang@connect.hku.hk (Y.P.); yuan1222@connect.hku.hk (Y.Y.); asterf@hku.hk (A.H.Y.F.)

**Keywords:** wet market, wooden cutting boards, surface hygiene, foodborne pathogens, nosocomial bacteria

## Abstract

Accessing food through wet markets is a common global daily occurrence, where fresh meat can be purchased to support an urbanizing world population. Similar to the wet markets in many other metropolitan cities in Asia, Hong Kong wet markets vary and are characterized by differing hygiene routines and access to essential modern technologies. The lack of risk assessments of food contact surfaces in these markets has led to substantial gaps in food safety knowledge and information that could help improve and maintain public health. Microbial profiling analyses were conducted on cutting boards that had been used to process pork, poultry, and seafood at 11 different wet markets. The markets differed in hygiene protocols and access to modern facilities. Irrespective of whether wet markets have access of modern infrastructure, the hygiene practices were largely found to be inefficient based on the prevalence of bacterial species typically associated with foodborne pathogens such as *Campylobacter fetus*, *Clostridium perfringens*, *Staphylococcus aureus*, and *Vibrio parahaemolyticus*; indicator organisms such as *Escherichia coli*; as well as nonfoodborne pathogenic bacterial species potentially associated with nosocomial infections, such as *Klebsiella pneumoniae* and *Enterobacter cloacae*. Other Vibrio species, *V. parahaemolyticus* and *V. vulnificus*, typically associated with contaminated raw or undercooked seafood with the potential to cause illness in humans, were also found on wooden cutting boards. This study indicated that the hygienic practices used in Hong Kong wet markets are not sufficient for preventing the establishment of spoilage or pathogenic organisms. This study serves as a basis to review current hygiene practices in wet markets and provides a framework to reassess existing safety protocols.

## 1. Introduction

Most people in developing countries still rely on wet markets to purchase their daily food supplies. Wet markets thus play a vital role in supplying fresh meat, vegetables, and fruits to the local population. Unfortunately, in some cases, wet markets have also had negative health implications. Earlier studies have indicated cross-contamination of cutting boards as a significant factor for foodborne outbreaks [1,2]. A recent study conducted in Brazil also showed that wooden cutting board surfaces were responsible for cross-contamination of foodborne pathogens from poultry to cucumber [3]. More specifically, cross-contamination of *Salmonella enterica* serovar Enteritidis occurred when processing cucumbers on cutting board surfaces previously used for poultry processing, irrespective of whether washing of the food contact surface was performed or not. Relatively less is known about cross-contamination and bacterial ecology in cutting boards from Asian wet markets. In our previous study, bacterial fingerprint assemblages associated with meat cutting board surfaces [4] identified pathogenic species such as *Klebsiella pneumoniae*, which is well known to cause nosocomial infections and harbor multidrug resistance [5]. The same study also showed the existence of antibiotic resistance genes in isolated bacterial strains [4]. A study conducted on meat sold in a Nepalese wet market reported the presence of foodborne pathogens including *Staphylococcus aureus*, *Escherichia coli*, *Salmonella* spp., *Pseudomonas* spp., *Vibrio* spp., and *Shigella* spp. [6]. It was revealed that the abundance of these foodborne pathogens was directly correlated with unhygienic processing, poor sanitation, and lack of food safety knowledge among the stallholders selling meat. Another study reported bacterial contamination of beef sold at wet markets in Malaysia [7]. In Hong Kong, a technical report indicated the presence of *Salmonella* on pork meat from a wet market [8] and in ready-to-eat meat products from different meat stalls [9].

Across the world, wet markets use cutting boards made of various materials, including wood and plastic. Although wooden cutting boards have been in use since time immemorial, they have only been considered to be hazardous surfaces since 1990. Wooden cutting boards harbor microbial contaminants and are difficult to clean. Moreover, wooden surfaces are porous and can be readily penetrated by bacteria, potentially leading to cross-contamination incidents [10].

The improper cleaning of wooden cutting board surfaces can promote the lodging of food debris in pores and crevices, which, in the presence of water, can lead to the development of microbial biofilms. The presence of such biofilms on wooden cutting boards can lead to an increased likelihood of cross-contamination during meat processing [11]. Moreover, biofilms also act as a protective barrier against sanitizers, thus increasing the likelihood of microbial survival or spoilage and pathogenic bacteria [12]. Biofilm formation by foodborne pathogens on food contact surfaces can lead to cross-contamination and thus compromise the safety of food processing [13,14]. *Salmonella* spp., *E. coli* O157:H7, *Vibrio parahaemolyticus*, *V. cholerae* O1, and *V. cholerae* O139 are the most commonly encountered foodborne disease-causing bacterial pathogens [15]. These pathogens can coexist in various foods, such as raw or undercooked meat [16]. A review of bacterial contaminants in poultry meat identified the presence of meat spoilage organisms such as *Pseudomonas fragi*, *P. lundensis*, and *P. fluorescens* [17]. In the family *Enterobacteriaceae*, the main bacterial species associated with poultry meat were *Hafnia alvei*, *H. paralvei*, *Serratia fonticola*, *S. grimesii*, *S. liquefaciens*, *S. proteamaculans*, *S. quinivorans*, *Rahnella* spp., *Yersinia* spp., and *Buttiauxella* spp. [17]. Bacterial species such as *Bacillus cereus*, *Clostridium botulinum*, *E. coli* O157, *V. cholerae*, and *V. parahaemolyticus* have also been linked to contaminated seafood [18,19,20].

Although Hong Kong’s wet markets have ramped up their hygiene awareness by implementing practical measures meant to promote food safety and safer food processing, a majority of existing wet markets continue to lack the much-needed modernization that allows for the proper storage and processing of foods. With food and environmental regulation committees regularly emphasizing the importance of safety standards in wet markets [21], it remains unclear whether guidelines designed for ensuring the cleanliness of wooden cutting boards are followed or effective. The goal of this study was, therefore, to provide an integrated view of the microbiome on wooden cutting boards used for the processing of pork, poultry, and seafood. The 11 wet markets in this study were selected based on their hygiene protocols and access to modern facilities. The microbial community on each cutting board was analyzed by pyrosequencing of the full-length region of the 16S ribosomal RNA gene using PacBio SMRT bell library preparation.

## 2. Material and Methods

### 2.1. Study Area and Sample Collection

**Figure 1:**
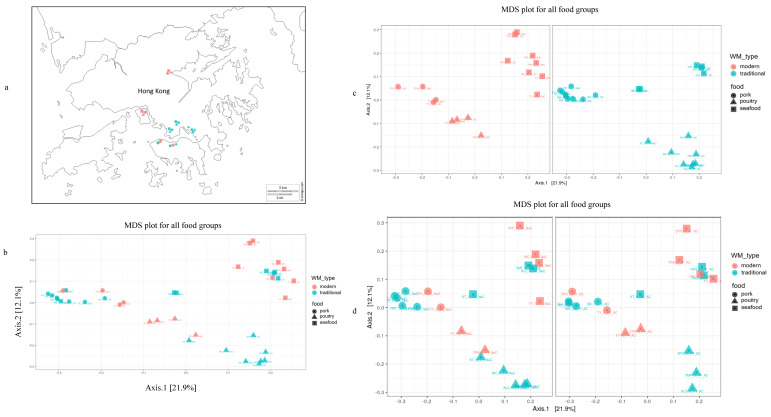
(**a**) Selected wet markets in Hong Kong based on their modern vs. traditional profiles; (**b**) Multidimensional scaling plot (MDS) of all samples from wooden cutting boards used for different food groups (pork, poultry, and seafood). (**c**) MDS plot for (i) modern wet market (left panel) and (ii) traditional wet market (right panel). (**d**) MDS plot for (i) uncleaned (left panel) and (ii) cleaned (right panel) cutting boards from different wet markets.

The sampling areas of this study were traditional or modern wet markets, situated in Kowloon City Market (KLC), Kwun Tung (KT), Ngau Tau Kok (NTK), Sai Ying Pun (SYP), Tai Po Hui (TPH), Wan Chai (WC), Tsing Yi (TY), and Yau Ma Tei (YMT). Traditional wet markets included outdoor shops or indoor markets without air conditioning. Modern wet markets were generally air-conditioned and typically located in buildings designated for wet market activities (Figure 1a). Swab samples were taken from wooden cutting boards that had been used for processing pork, poultry, and seafood products in the markets. To investigate the efficacy of cleaning protocols adopted for cutting boards from individual wet market stalls, the samples were collected before and after cleaning of the cutting boards. The meat stallholders commonly used the following cleaning methods: (i) scraping the entire surface of the cutting board, (ii) rinsing the cutting board with cold water, (iii) rinsing the cutting board with warm water, and (iv) rinsing the cutting board with detergents (Supplementary Table S1). Our previous work provides a detailed description of the scrapping method used in wet markets [4].

All sampling procedures were performed with the consent of the stall owner. It should be noted that not all selected wet markets allowed successful sampling from pork-, poultry-, and seafood-designated cutting boards either due to unavailability of the boards or due to individual stall owners not consenting to participate in the study. There were 39 wooden cutting boards sampled from 11 different wet markets in total: 13 for pork processing, 12 for poultry processing, and 14 for seafood processing. All swab samples were collected in July 2019.

The swabs (Zymo, CA, USA) were used to sample from an area of ~18 cm × 8 cm on the boards, as previously described by Lo et al. [4], with slight modifications. The collected swab samples were preserved in DNA/RNA Shield collection tubes (R1107, Zymo) at room temperature. The Shield solution (Zymo) allows the preservation of sampled DNA for up to 1 year at room temperature. The total genomic DNA (gDNA) from each sample was extracted within one month of sampling.

### 2.2. DNA Extraction and Sequencing

ZymoBIOMICS DNA Miniprep D4300 (Zymo) and D4301 (Zymo) were used to extract gDNA from swab samples obtained from uncleaned and cleaned cutting boards, respectively. The samples preserved in Shield solution were transferred to bead-beating tubes provided in the extraction kits. Bead-beating was performed at 30 Hz for 8 min using a TissueLyser II (Qiagen, Venlo, The Netherlands). All centrifugations were performed in a D2012 Plus mini centrifuge (Scilogex, Rocky Hill, CT, USA). All extraction processes were performed using the standardized method suggested by the manufacturer. The extracted DNA samples were stored at −80 °C until further usage to avoid DNA degradation. Before sequencing, the optical density values and DNA concentrations of the extracted and purified DNA samples were examined using a BioDrop µLITE (BioDrop, Cambridge, UK) to determine the quality and purity of DNA. DNA degradation was assessed by gel electrophoresis. All gDNA samples were sent to the Centre for PanorOmic Sciences (The University of Hong Kong, Hong Kong) for PacBio Sequencing.

Amplicon sequencing was performed using the universal primer pair 27F: AGRGTTYGATYMTGGCTCAG and 1492R: RGYTACCTTGTTACGACTT to amplify the full-length 16S rRNA genes from the gDNA samples. The KAPA HiFi HotStart DNA Polymerase (KAPA Biosystems, Wilmington, MA, USA) was used to perform 20 cycles of polymerase chain reaction (PCR) amplification under the following conditions: denaturing at 95 °C for 30 s, annealing at 57 °C for 30 s, and extension at 72 °C for 60 s. Postamplification quality control was performed using a Bioanalyzer (Agilent Technologies, Santa Clara, CA, USA). SMRTbell libraries were prepared from the amplified DNA by blunt-end ligation according to the manufacturer’s instructions (Pacific Biosciences, Menlo Park, CA, USA). The purified SMRTbell libraries generated from the DNA samples were sequenced on dedicated PacBio Sequel cells using S/P1-C1.2 sequencing chemistry.

### 2.3. Sequencing Data Analysis

The Divisive Amplicon Denoising Algorithm (DADA2) [22] was used to infer the amplicon sequence variants (ASVs) that differed from each other by at least one nucleotide. The ASVs were inferred from the filtered reads using the DADA2 R-software package version 1.12.1 that has been updated to efficiently process long amplicon reads and appropriately model PacBio CCS sequencing errors. DNA samples from the 39 wooden cutting boards were assessed, and microbial community profiling was performed based on the access of wet markets to modern amenities (i.e., traditional vs. modern) and their hygiene protocol (i.e., uncleaned vs. cleaned).

### 2.4. Algorithm and Bioinformatic Analysis

Circular consensus sequence (CCS) reads were generated from the raw PacBio amplicon sequencing data of the swab samples using the CCS application with minPasses = 3 and minPredictedAccuracy = 0.999 in SMRT Link 3.1.1 software (Pacific Biosciences). The DADA2 R package tool was used to remove and orient primers from CCS reads. The filterAndTrim function in the DADA2 method was used to determine the ASVs (i.e., the true error-free sequences) present in a sample among the library of noisy reads generated by amplicon sequencing. Error models were used in DADA2-R to identify sequences that were repeatedly observed too many times to be consistent with being generated by amplicon sequencing errors. Chimeras were also removed to generate an error-corrected table of the abundances of ASVs in each sample as the output.

### 2.5. Filtering of Pathogens in the Microbial Community

The resulting ASVs were processed using R packages including phyloseq [23], DESeq2 [24], and ggplot2 [25] or with downstream analysis and visualization including ordination, alpha/beta diversity calculations, and relative abundances. Sequence data were screened for the presence of pathogens using existing literature on foodborne and clinical pathogens. Relative ASV abundance of identified pathogens was then calculated based on cutting board food type (used for pork, poultry, or seafood processing) or state of cleanliness (cleaned/uncleaned).

## 3. Results

### 3.1. Bacterial Diversity Indices in Swab Samples from Wet Market Cutting Boards

The PacBio full-length 16S rRNA gene sequencing data was used to analyze the alpha diversity in the cutting board swab samples from the 39 wet markets. The Shannon and Simpson diversity indices and species estimates showed higher biodiversity metrics on cutting boards used for seafood processing followed by boards used for poultry and pork processing (Supplementary Figure S1). Biodiversity estimation was also compared between market types (Supplementary Figure S1a). Across all food types, the Shannon diversity index was higher for traditional markets than for modern ones. Higher diversity was generally observed in samples from boards used for seafood, followed by samples from boards used for poultry and pork in both traditional and modern market types. To test the effect of the cleanliness state of cutting boards on microbial diversity, we estimated diversity indices of samples obtained before and after cleaning. The bacterial diversity was higher on uncleaned cutting boards used for processing pork and seafood (Supplementary Figure S1b). The bacterial diversity of cutting boards used for poultry processing did not differ before and after cleaning. Only those used for pork and seafood processing had decreased bacterial diversity following washing procedures.

### 3.2. Multidimensional Scaling and Microbial Structures of Bacterial Communities

The variability of bacterial communities on cutting boards used for pork, poultry, and seafood processing was investigated using multidimensional scaling of the Unifrac distance matrix. The microbial communities were clustered based on food types (Figure 1b). In samples from both traditional and modern wet markets, the same food group clustering’s were observed (Figure 1c). The poultry and pork samples were tightly clustered in modern wet market samples but were separated in traditional wet markets (Supplementary Figure S2a).

Uncleaned cutting boards from modern and traditional markets also had distinguishable subclusters for all food types (Supplementary Figure S2b). This finding suggests that the intrinsic microbiological load on the sampled cutting boards differed due to improved hygiene protocols in modernized wet markets. Washed cutting boards from modern and traditional wet markets had different subclusters within the main poultry cluster but not within the pork or seafood clusters (Figure 1d).

### 3.3. Bacterial Diversity on Wooden Cutting Boards Used for Different Food Types

**Figure 2:**
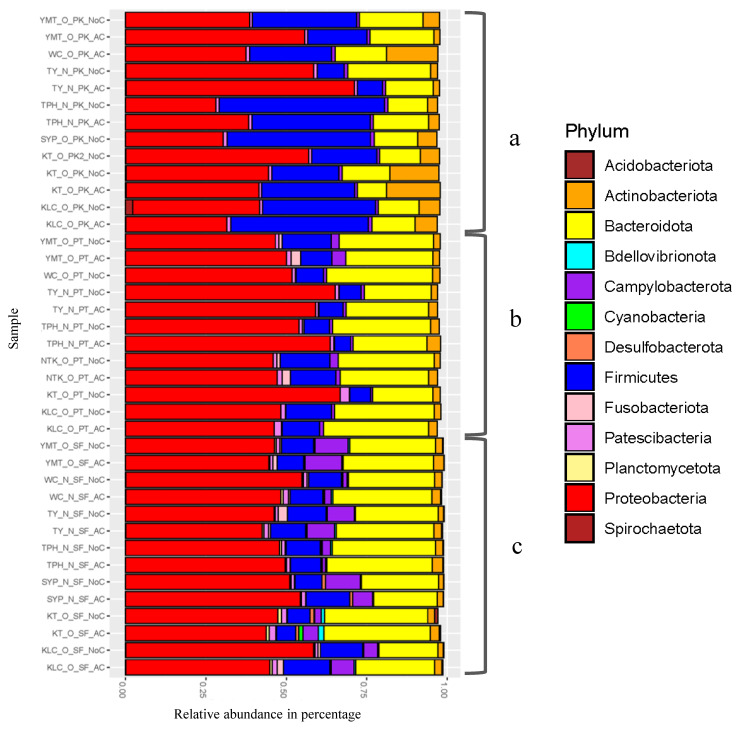
Taxonomic profile: Relative phylum-level abundance of bacteria in samples from wooden cutting boards used for different food groups: (**a**) pork, (**b**) poultry, and (**c**) seafood.

The 16S metagenomic amplicon full-length sequencing data of swab samples collected from the 11 wet markets showed that the three most abundant phyla commonly found on cutting boards used for pork, poultry, and seafood processing were Proteobacteria, Firmicutes, and Bacteroidota (Figure 2). The dominant families on cutting boards used for pork processing included *Staphylococcaceae*, *Moraxellaceae*, *Flavobacteraceae*, and *Mycobacteriaceae*; for poultry processing, they included *Moraxellaceae*, *Weeksellaceae*, *Burkholderiaceae*, and *Neisseriaceae*; and for seafood processing, they included *Acrobacteriaceae*, *Burkholderiaceae*, *Moraxellaceae*, and *Weeksellaceae* (Supplementary Figure S3).

#### Meta-Analysis of Bacterial Community Composition on Wooden Cutting Boards

On cutting boards used for pork processing, the dominant genera in samples from traditional wet markets were *Macrococcus* and *Corynebacterium* (Supplementary Figure S4a), while those found in samples from modern wet markets included *Acinetobacter* and *Staphylococcus*. The bacterial genera found in samples from poultry cutting boards used in modern wet markets were less diverse than those found in samples from traditional wet markets. *Acinetobacter* and *Empedobacter* were the most abundant genera on cutting boards from modern wet markets, but *Moraxella* was the most abundant genus from traditional wet markets (Supplementary Figure S4b). No difference was found in the genus-level representation between modern and traditional wet markets for seafood cutting boards (Supplementary Figure S4c).

To assess the impact of hygiene on cutting boards, we analyzed the top 50 genera in the sequencing data before and after cleaning the cutting boards. Changes in genus-level abundance were observed following washing routines of cutting boards. In one wet market (TPH), the washing of cutting boards led to the appearance of *Myroides* and *Aeromonas*, while increasing the abundance of *Brochothrix* and *Acinetobacter* (Supplementary Figure S5a). When hygiene maintenance did not involve the use of cleaning detergent, the abundance of *Vitreoscilla* and *Chryseobacterium* increased (Supplementary Figure S5c) but was not influenced by the modernity of the wet market (Supplementary Table S1). In contrast, the washing of cutting boards used in the processing of poultry generally led to a reduced bacterial abundance (Supplementary Figure S5b). In wet markets, the cleaning procedures seemingly removed *Empedobacter* (YMT), *Comamonas* and *Moraxella* (TPH), and *Moraxella* (NTK) (Supplementary Figure S5c). Both the YMT and TPH wet markets used the most stringent cleaning methods (Supplementary Table S1). The absence of *Marinomonas* (YMT), *Aeromonas* and *Brachymonas* (TPH), and *Cloacibacterium* (KLC) following cleaning routines used on cutting boards meant for seafood processing suggests some degree of efficiency in the removal of spoilage organisms. It is also notable that the YMT, TPH, and KLC wet markets used the same cleaning method as shown in Supplementary Table S1.

### 3.4. Detection of Putative Pathogens on Wooden Cutting Boards

**Figure 3:**
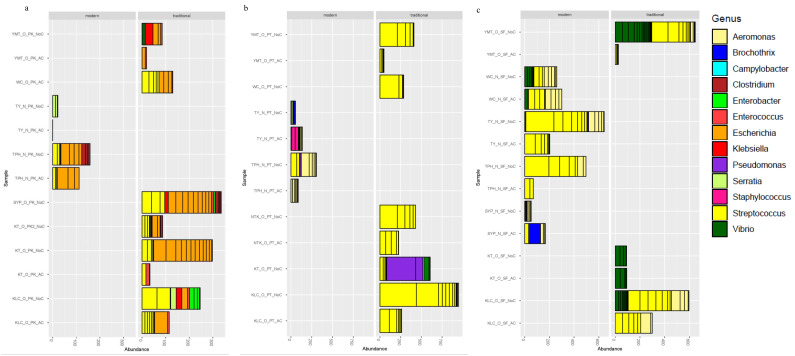
The relative genus-level abundance of bacteria in samples from wooden cutting boards used for different food groups: (**a**) pork, (**b**) poultry, and (**c**) seafood. Left (i) samples collected in modern wet markets. Right (ii) samples collected in traditional wet markets.

Further analysis was conducted to determine the pathogenic microorganisms found on the cutting boards. Pathogens commonly found in contaminated pork, poultry, and seafood were extracted and are shown in representative plots (Figure 3).

#### Microbial Profiles of Cutting Boards Were Differentiated by the Type of Processed Foods

The cutting boards used for pork processing harbored the most bacterial species associated with either food pathogens or food spoilage organisms compared to the other boards. Various foodborne bacterial species such as *Campylobacter fetus*, *Clostridium perfringens*, *Enterobacter cloacae*, *Enterococcus* spp., *Escherichia coli*, *Klebsiella pneumoniae*, *Pseudomonas putida*, *Serratia marcescens*, *Streptococcus suis*, and *Vibrio* spp. were specifically identified from cutting boards used for pork processing. The most common bacterial species known to be emerging pathogens or food spoilage organisms on boards used for poultry were *Aeromonas* spp., *Brochothrix campestris*, *Enterobacter clocae*, *Pseudomonas putida*, *Serratia marscescens*, *Staphylococcus aureus*, and *Streptococcus* spp. Seafood-processing boards harbored *Aeromonas dhakensis*, *Brochothrix campestris*, *Streptococcus* spp., *V. vulnificus*, and *V. parahaemolyticus*.

### 3.5. Microbial Profiles of Cutting Boards Were Differentiated by the Type of Wet Markets and Recorded Hygiene Practices

**Figure 4:**
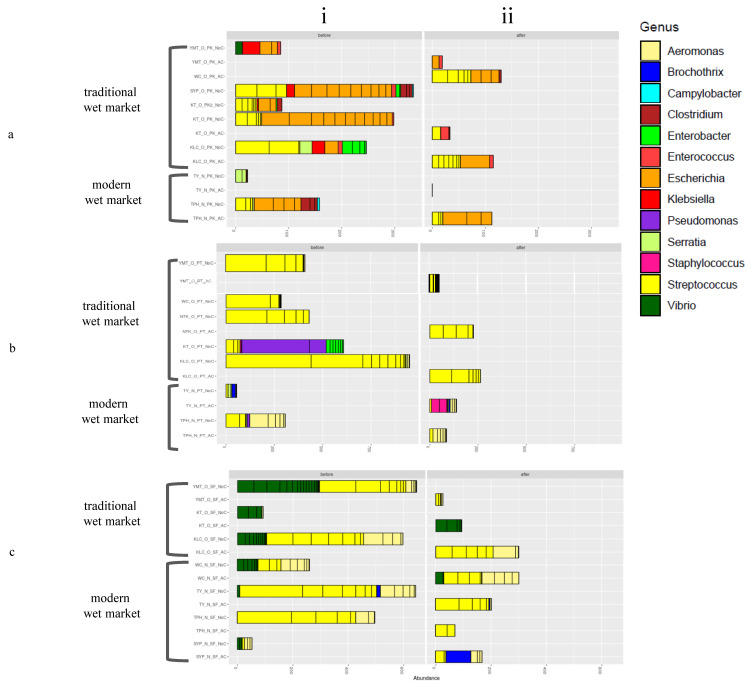
The relative genus-level abundance of bacteria in samples from wooden cutting boards used for different food groups: (**a**) pork, (**b**) poultry, and (**c**) seafood. Left (i) before cleaning, and (ii) after cleaning.

On pork-processing cutting boards, *Escherichia* and *Streptococcus* were the most abundant genera. *Klebsiella*, *Vibrio*, and *Enterobacter* were the most abundant genera in traditional wet markets (Figure 3a). The cutting boards from traditional wet markets also had a high abundance of the top 50 genera compared to those from modern wet markets. *Klebsiella*, *Vibrio*, and *Enterobacter* were the most abundant genera in traditional wet markets (Figure 4). A higher abundance and diversity of pathogens were observed on uncleaned cutting boards used for pork processing than on cleaned cutting boards. The *Enterobacter*, *Clostridium*, and *Serratia* genera were commonly found on uncleaned cutting boards. A higher abundance and diversity of these common pathogenic genera were found on uncleaned cutting boards than on cleaned cutting boards, suggesting, as expected, that the cleaning procedure reduced the pathogenic load on the cutting boards (Figure 3a).

*Streptococcus* was the most abundant genus on cutting boards used for poultry processing. Samples from modern wet markets had a high abundance of the *Aeromonas* genus, while those from traditional wet markets had a high abundance and diversity of the *Streptococcus* genus. The abundance and diversity of *Streptococcus* were considerably lower in modern wet markets than in traditional wet markets (Figure 3b). As expected, uncleaned cutting boards had a higher abundance of several pathogenic genera than did clean cutting boards. At one market (TY), washed cutting boards contained *Staphylococcus* (Figure 3b), suggesting that the cleaning technique of only rinsing with cold water and no detergent was ineffective (Supplementary Table S1) and likely caused cross-contamination. However, in most other markets, washing the cutting boards reduced the bacterial load (Figure 3).

On seafood-processing cutting boards, *Aeromonas*, *Streptococcus*, and *Vibrio* were the most abundant genera. Only *Brochothrix* was found on cutting boards from modern wet markets (Figure 3c). The highest diversity of *Vibrio* was identified on cutting boards from wet markets close to hospitals (Figure 4c). As expected, uncleaned cutting boards had a higher abundance of several pathogenic genera than cleaned cutting boards did. At the WC and KT wet markets, washed cutting boards had the same abundance or composition of pathogenic bacteria (Figure 3c). At the SYP market, washed cutting boards had *Brochothrix*, suggesting that the cleaning technique was ineffective and likely caused cross-contamination. In most other markets, washing cutting boards reduced the bacterial load.

## 4. Discussion

This study demonstrated measurable variations among bacterial populations on wooden cutting boards used for different food types (pork, poultry, and seafood) and sourced from different types of wet markets (traditional vs. modern). The food type was a critical factor that shaped the bacterial community structure on the cutting boards. The comparison between samples from traditional and modern wet markets also showed trends in diversity indices and had different bacterial profiles. Traditional wet market samples had higher diversity and abundance of bacterial assemblages, which are known to be either food spoilage or food pathogen organisms, in comparison to modern wet market samples. As expected, cleaning cutting boards led to a significant reduction in bacterial abundance.

Traditional wet market samples had distinct bacterial species, such as *Acinetobacter baumannii*, *Acinetobacter nosocomialis*, *Aeromonas jandei*, *Aeromonas dhakensis*, *Bacillus cereus*, *Klebsiella pneumoniae*, *Enterobacter cloacae*, *Staphylococcus aureus*, and *Staphylococcus epidermidis* (Table 1), and a high abundance of other bacterial species (Supplementary Figure S6). The low bacterial diversity found in modern wet market samples is likely attributable to the controlled environmental conditions (e.g., air conditioning and low relative air humidity), meant to reduce the proliferation of bacterial pathogens, as has been reported in similar previous studies [4]. In contrast, traditional wet market vendors usually process their meat in uncontrolled conditions (e.g., high temperatures and high relative air humidity), which may promote the proliferation of various pathogens. Earlier studies revealed a diversity caveat, in which microbial diversity was found to be directly linked to ecosystem functioning. More specifically, microbial communities with greater microbial diversity performed better ecosystem functions [26]. Another study on plant root rhizosphere diversity revealed that complex microbial consortia featuring a high diversity of *Pseudomonas* species were able to suppress the persistence of soilborne pathogens, hence suggesting that increased diversity may also disfavor the survival of pathogens [27]. It should also be noted that reduced diversity could also favor the survival of pathogens in modern wet markets even though the overall load is reduced. Therefore, it could be argued that cutting boards from “traditional settings” may actually be safer, based on their higher microbial diversity profiles, thereby suggesting a need to reevaluate the potential risks arising out of microbiomes of modern wet market environments. However, on the basis of a zero-tolerance policy, the presence of pathogenic organisms in both traditional or modern cutting boards should indicate a significant breach in hygiene procedures meant for guaranteeing food safety, and ultimately, public health.

The detection of *Klebsiella pneumoniae* and *Enterobacter cloacae* in traditional wet markets should not be ignored, as these species are typically associated with many nosocomial infections and are associated with multidrug resistance and good biofilm formation [28,29]. Microorganisms with such biofilm-forming capabilities also hint at their ability to survive, persist, and thrive in traditional wet market environments. Cleaning measures adopted in both traditional and modern wet markets did not eradicate *Brochothrix campestris*, a species typically associated with meat spoilage [30,31,32]. This finding suggests that the cleaning and preservation techniques used in traditional and modern wet markets are not sufficient for preventing the establishment of spoilage organisms. Furthermore, washing the cutting board in one wet market (TPH) has led to a dominance of *Aeromonas* species, often associated with aquatic environments [33,34,35] and known to be good biofilm formers [33]. Several studies have pointed out the pathogenicity and antimicrobial resistance of the *Aeromonas* species in aquatic environments [34,35]. The sudden appearance of *Aeromonas* in this context suggests poor water quality or poor sanitation equipment used, such as the use of dirty sponges or towels, during the cleaning of the cutting board. Based on the pathogenic risks of bacterial species, this study calls for the monitoring of the following species in Hong Kong, based on wet market type (i.e., modern vs. traditional wet market) and hygienic practices, as shown in Table 1.

Samples from pork-processing cutting boards contained *Campylobacter*, a universal diarrhea-causing pathogen. The presence of *Campylobacter* on sampled cutting boards is significant, as species such as *C. jejuni* and *C. coli* account for nearly 90% and 10% of reported foodborne illnesses and foodborne humans’ infections, respectively [36]. A previous technical report on Hong Kong’s wet markets also revealed the microbiological hazards of pork meat and lack of food hygiene [8]. However, we still observed a lack of hygiene and detected several foodborne pathogens on the wooden cutting boards. Infection with *Streptococcus suis*, an emerging pathogen, is acquired through exposure to contaminated pigs or pig meat [37]. In this study, a species-level pathogen analysis indicated the presence of *Streptococcus suis* on cutting boards used for pork processing (Table 1). Other bacteria harmful to human health were also identified, e.g., *Klebsiella pneumoniae*, which is known to cause pneumonia [38]. *Staphylococcus aureus* and *Clostridium perfringens*, which are associated with other foodborne illnesses [32], and *Bacillus cereus*, a foodborne pathogen known for its spore-forming abilities, were also found. *Bacillus cereus* can survive several environmental stresses and causes a foodborne illness with symptoms of diarrhea, vomiting, and abdominal pain [39]. An earlier study, which addressed the microbiological quality of meat from local Hong Kong markets, revealed the presence of *Salmonella* species and absence of *Campylobacter* species in sampled pork meat [8]. Conversely, this study indicated the presence of *Campylobacter* and absence of *Salmonella* species in wet market cutting boards. It is worth noting that the Center for Food Safety and Food and Environmental Hygiene Department in Hong Kong has implemented procedures to reduce *Salmonella* in pork meat, which may explain its low detection in this study.

The pathogenic bacterial species found on pork-processing boards acquired from markets were identified as *Klebsiella pneumoniae*, *Vibrio parahaemolyticus*, and *Enterobacter cloacae*. The presence of these organisms should be alarming and reflects an insufficient level of food safety and risk to public health. A previous study reported the presence of bacteria containing antibiotic resistance genes on cutting board surfaces [4], which also included nosocomial pathogens (Table 1) such as *Acinetobacter baumannii*, *Acinetobacter nosocomialis*, *Klebsiella pneumonia*, and *Serratia marcescens*. The prevalence of pathogens in cutting boards may be an indicator of the sample’s previous handling and the level of hygiene at the point of purchase. Typically, supermarkets also process their meat products using wooden cutting boards before packaging and storing, usually under controlled temperature and relative humidity conditions. In sharp contrast, wooden cutting boards from wet markets are exposed to a combination of Hong Kong’s high ambient temperature and high relative humidity conditions and are conducive to pathogenic microbial proliferation on freely displayed meat products [5].

The bacterial assemblages based on the 16S rRNA full-length sequencing data showed higher bacterial abundance, as reflected by the diversity indices (Supplementary Figure S1). The high abundance of bacterial pathogens was primarily associated with uncleaned cutting board surfaces. According to Hong Kong’s Food and Environmental Hygiene Department, food contact surfaces of equipment used for processing foods should (i) be made of a nontoxic, nonabsorbent, and noncorrosive smooth material; (ii) remain unaffected by grease, food particles, or water; (iii) be free from cracks, crevices, and open seams; (iv) allow for complete cleaning and sanitization; and (v) be easy to clean, sanitize, and inspect. Unfortunately, there are no established guidelines on the methods for maintaining wooden cutting boards in Hong Kong’s wet markets. Of the four common cleaning methods listed in Supplementary Table S1, most stall-keepers rely on only scraping the top layer without additional cleaning with disinfectants. Although the scraping method and cold-water rinse were effective in reducing pathogen loads on pork contact surfaces, this method led to the appearance of *Staphylococcus* in TY market sample on poultry contact surfaces. On seafood contact surfaces, the scraping method led to the appearance of *Brochothrix campestris*, even after using detergents. Several studies have suggested that disinfectants can also lead to the development of disinfectant-resistant bacteria and might not eliminate some antibiotic-resistant bacteria [40]. This idea raises serious concerns over the hygiene practices performed in wet markets and warrants the implementation of a strict protocol for the routine disinfection of meat-processing cutting boards.

The present study provides more information about the hygiene of cutting boards used in Hong Kong’s wet markets. Pinpointing the exact or presumed origin of identified pathogens remains a significant challenge in tracing the sources of potential cross-contamination in such environments. Future epidemiological research in which nonfoodborne pathogenic isolates from environmental samples are compared with isolates from clinical settings is therefore needed. It is only in this context that a direct link can be established between the presence of presumptive nonfoodborne pathogens isolated from wet market cutting boards with clinical environments via cross-contamination.

In developed countries such as those in Europe, materials used as food contact surfaces are subject to European regulation (EC) No 1935/2004, which states that the materials must not transfer their constituents to food [41]. Our bacterial analysis on wooden cutting board surfaces used in wet markets showed the presence of pathogens even after detergent washing. A previous study detected a high diversity of bacteria on kitchen cutting boards made of plastic and wood [42]. Another disadvantage of using wood as a food contact surface material is that its porosity allows the formation of biofilms on the surfaces [43,44], which are difficult to disinfect. Our analysis indicated that the surface scraping method only reduced the microbial diversity on the surface, as biofilm-forming bacteria remained in the pores and crevices of the cleaned cutting boards.

## 5. Conclusions

In this study, bacterial profiling was performed on the surfaces of cutting boards used for processing pork, poultry, and seafood in Hong Kong’s wet markets. Foodborne and nonfoodborne pathogens were present on cutting board surfaces. Although the exact transmission routes of these pathogens could not be fully determined, cross-contamination events in wet markets might explain the possible spread of foodborne and nonfoodborne pathogens. Current methods for the control of specific spoilage-related genera in wet market settings are still lacking. This study indicated that the hygienic practices used in Hong Kong wet markets are not effective for preventing the establishment of spoilage or pathogenic organisms. This study should, therefore, serve as a basis to review the current hygiene practices in wet markets in both Asia and worldwide to improve food safety and, ultimately, public health.

## Figures and Tables

**Table 1:** Bacterial species on wooden cutting board surfaces in Hong Kong wet markets.

	Wet Market Type		Cutting Board Hygiene/Cleaning		
Cutting Board Associated Bacterial Species	Traditional	Modern	Before	After	Food Type
*Acinetobacter baumannii*	YES	YES	YES	YES	pork
*Acinetobacter nosocomialis*	YES	YES	YES	YES	pork
*Aeromonas dhakensis*	YES	YES	YES	YES	pork, seafood
*Aeromonas jandei*	YES	NO	YES	NO	pork
*Bacillus cereus*	YES	NO	YES	NO	pork
*Brochothrix campestris*	YES	YES	YES	YES	pork, poultry, seafood
*Campylobacter* spp.	YES	YES	YES	YES	pork
*Clostridium perfringens*	YES	YES	YES	YES	pork
*Enterobacter cloacae*	YES	NO	YES	NO	pork, poultry
*Escherichia coli*	YES	YES	YES	YES	pork
*Enterococcus faecalis*	NO	YES	YES	YES	pork
*Klebsiella pneumoniae*	YES	NO	YES	NO	pork
*Pseudomonas putida*	YES	YES	YES	YES	pork, poultry
*Staphylococcus epidermidis*	YES	NO	YES	NO	pork
*Staphylococcus aureus*	YES	NO	YES	NO	poultry
*Streptococcus suis*	YES	YES	YES	YES	pork
*Serratia marcescens*	YES	YES	YES	YES	pork, poultry
*Vibrio vulnificus*	YES	YES	YES	NO	seafood
*Vibrio parahaemolyticus*	YES	YES	YES	YES	seafood

## Data Availability

Raw sequencing reads have been deposited in the EMBL-EBI Sequence Read Archive under the accession number PRJEB37431.

## Supplementary Materials

The following are available online at https://www.mdpi.com/2076-2607/8/12/1941/s1. Supplementary Figure S1. Alpha diversity comparing by (a) wet market type; (b) cleaning conditions; Supplementary Figure S2. Multidimensional scaling (MDS) plot of samples separated by (a) wet market type, (i) modern wet market and (ii) traditional wet market; (b) cleaning practice, (i) before cleaning and (ii) after cleaning. Supplementary Figure S3. The relative abundance of top 20 families sampled from wooden cutting board processed for (a) pork, (b) poultry, or (c) seafood processing cutting board. Supplementary Figure S4. The relative abundance of top-50 genus sampled from wooden cutting board processed for (a) pork, (b) poultry, and (c) seafood. Left (i) represents samples from the modern wet market. Right (ii) represents samples from the traditional wet market. Supplementary Figure S5. The relative abundance of top-50 genus sampled from wooden cutting board processed for (a) pork, (b) poultry, and (c) seafood. Left (i) represents samples before practicing cleaning. Right (ii) represents samples after practicing cleaning. Supplementary Figure S6. The relative species-level abundance of bacteria in samples from wooden cutting boards: (a) traditional vs. modern, (b) cleaned vs. uncleaned, (c) different food groups (pork, poultry, seafood). Supplementary Table S1: Common cutting board cleaning practices in traditional and modern wet markets of Hong Kong.
