# Supplementary material for: Impacts of Wet Market Modernization Levels and Hygiene Practices on the Microbiome and Microbial Safety of Wooden Cutting Boards in Hong Kong
Source: Microorganisms. 2020 Dec 7;8(12):1941. doi: 10.3390/microorganisms8121941 (PMC7762345; doi:10.3390/microorganisms8121941)
Supplement: Supplementary file 1 [file microorganisms-08-01941-s001.zip › microorganisms-1030018-supplementary/MDPI_Supplementary_Fig.4.pdf]

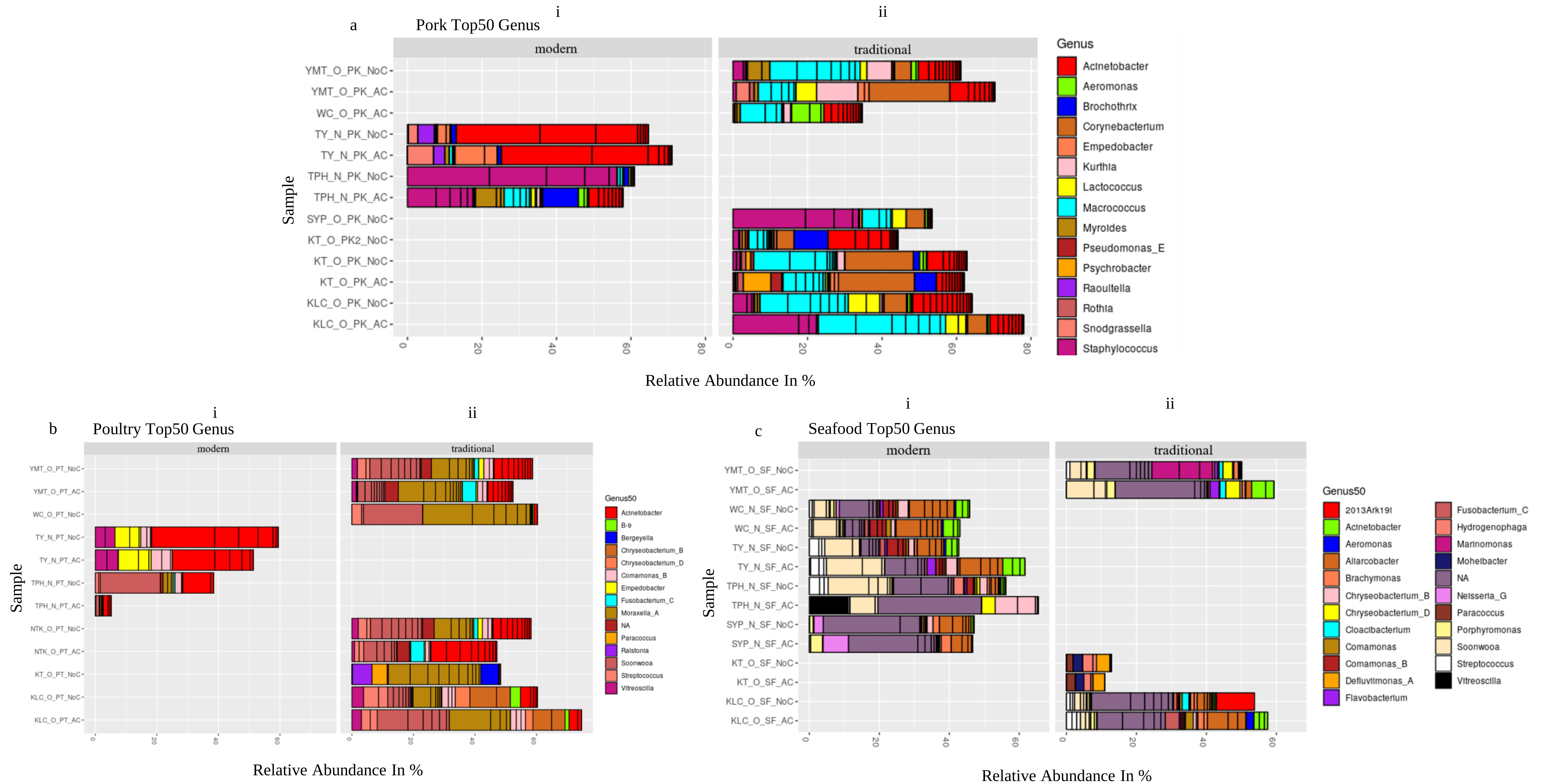

Supplementary Fig. 4. Relative abundance of top-50 genus sampled from wooden cutting board processed for a) pork; b) poultry and c) seafood. Left (i) represents samples from modern wet market. Right (ii) represents samples from traditional wet market.
