# Supplementary material for: Impacts of Wet Market Modernization Levels and Hygiene Practices on the Microbiome and Microbial Safety of Wooden Cutting Boards in Hong Kong
Source: Microorganisms. 2020 Dec 7;8(12):1941. doi: 10.3390/microorganisms8121941 (PMC7762345; doi:10.3390/microorganisms8121941)
Supplement: Supplementary file 1 [file microorganisms-08-01941-s001.zip › microorganisms-1030018-supplementary/MDPI_Supplementary_Fig.6.pdf]

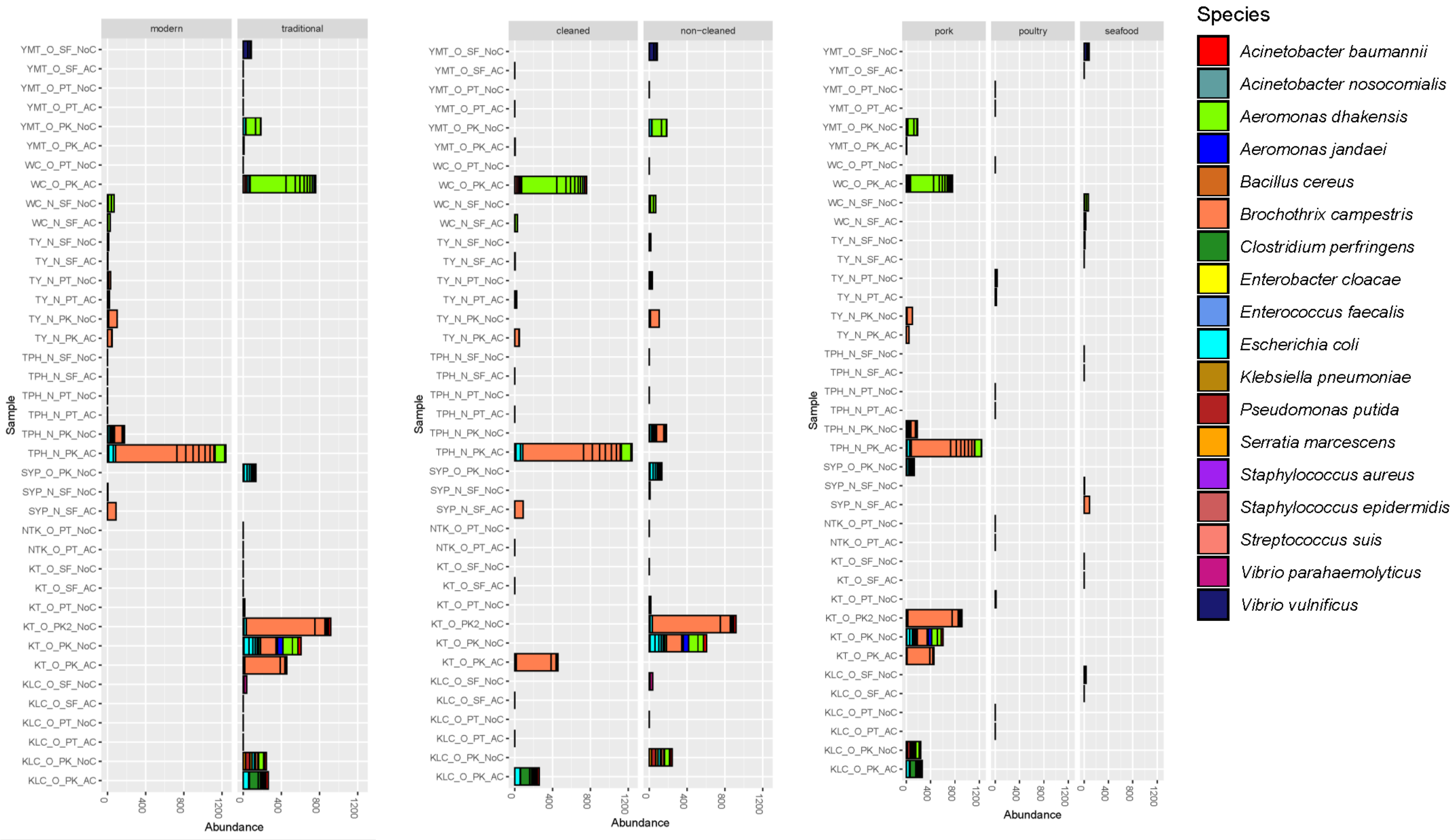

Supplementary Fig. 6. Relative species-level abundance of bacteria in samples from wooden cutting boards: a) traditional Vs modern, b) cleaned Vs non-cleaned c) different food groups (pork, poultry, seafood).
