# Supplementary figures and images for: Impacts of Wet Market Modernization Levels and Hygiene Practices on the Microbiome and Microbial Safety of Wooden Cutting Boards in Hong Kong
Source: Microorganisms. 2020 Dec 7;8(12):1941. doi: 10.3390/microorganisms8121941 (PMC7762345; doi:10.3390/microorganisms8121941)

a

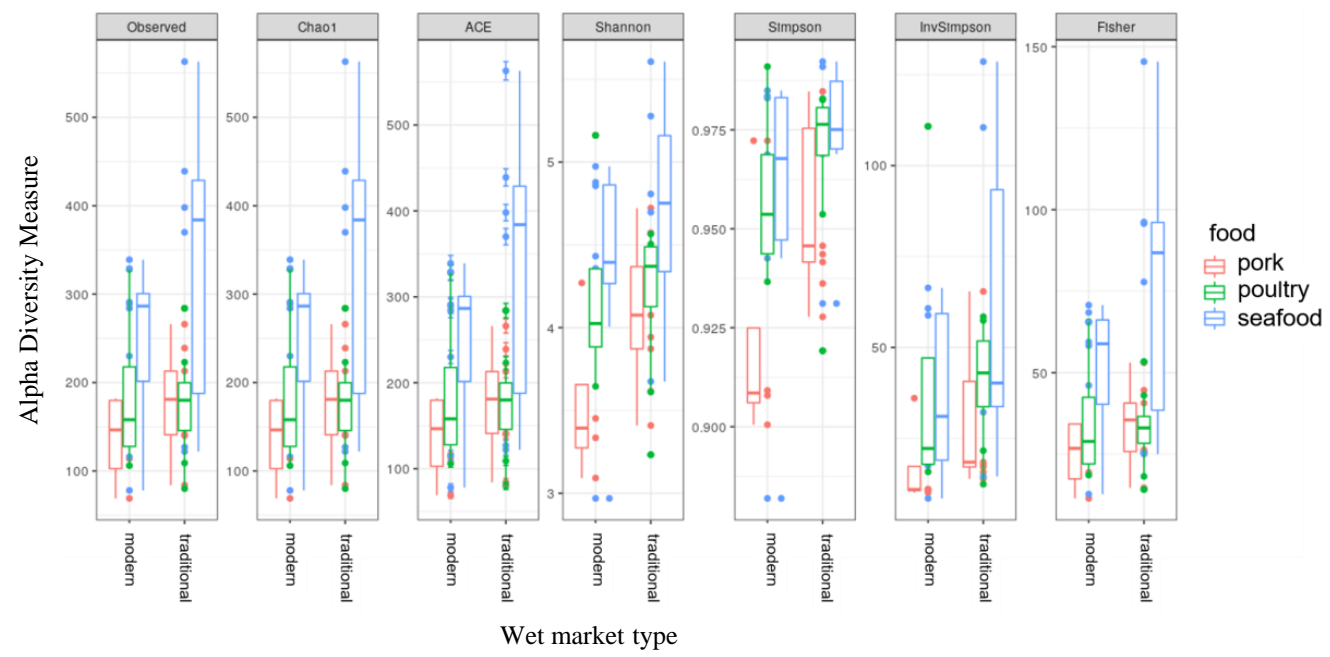

b

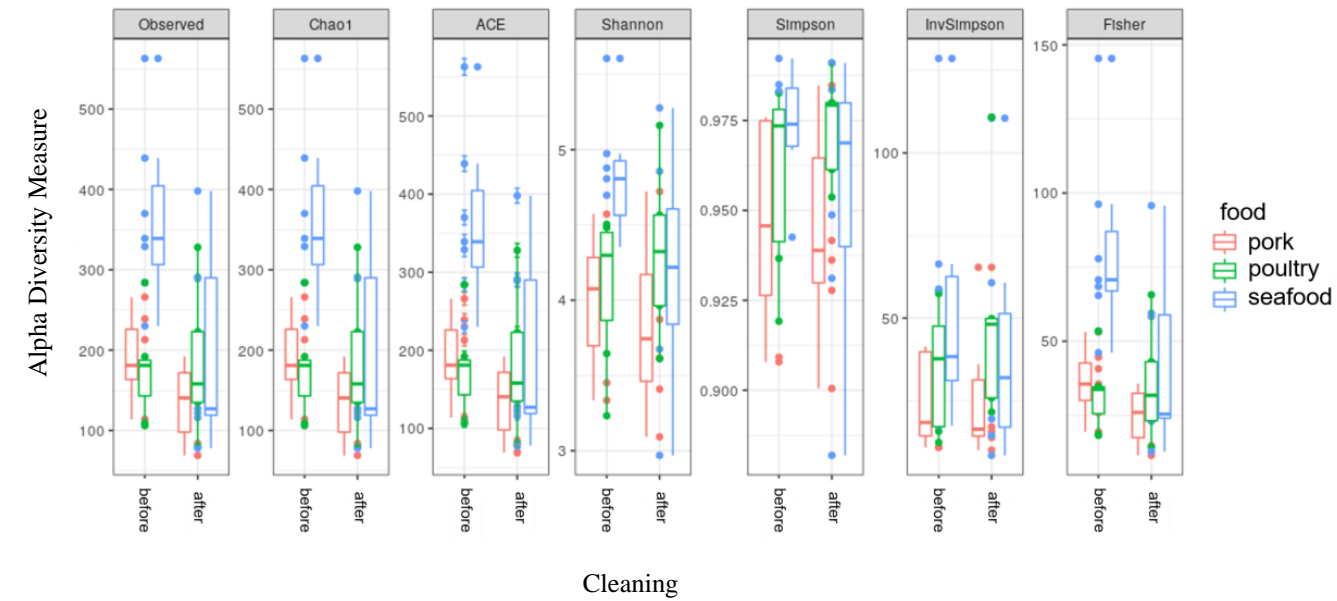

Supplementary Fig 1. Alpha diversity comparing by (a) wet market type; (b) cleaning conditions.

Supplement: Supplementary file 1 [file microorganisms-08-01941-s001.zip › microorganisms-1030018-supplementary/MDPI.Supplementary.Fig.1.pdf]
